# Supplementary figures and images for: Evaluating fairness of machine learning prediction of prolonged wait times in Emergency Department with Interpretable eXtreme gradient boosting
Source: PLOS Digit Health. 2025 Mar 20;4(3):e0000751. doi: 10.1371/journal.pdig.0000751 (PMC11925291; doi:10.1371/journal.pdig.0000751)

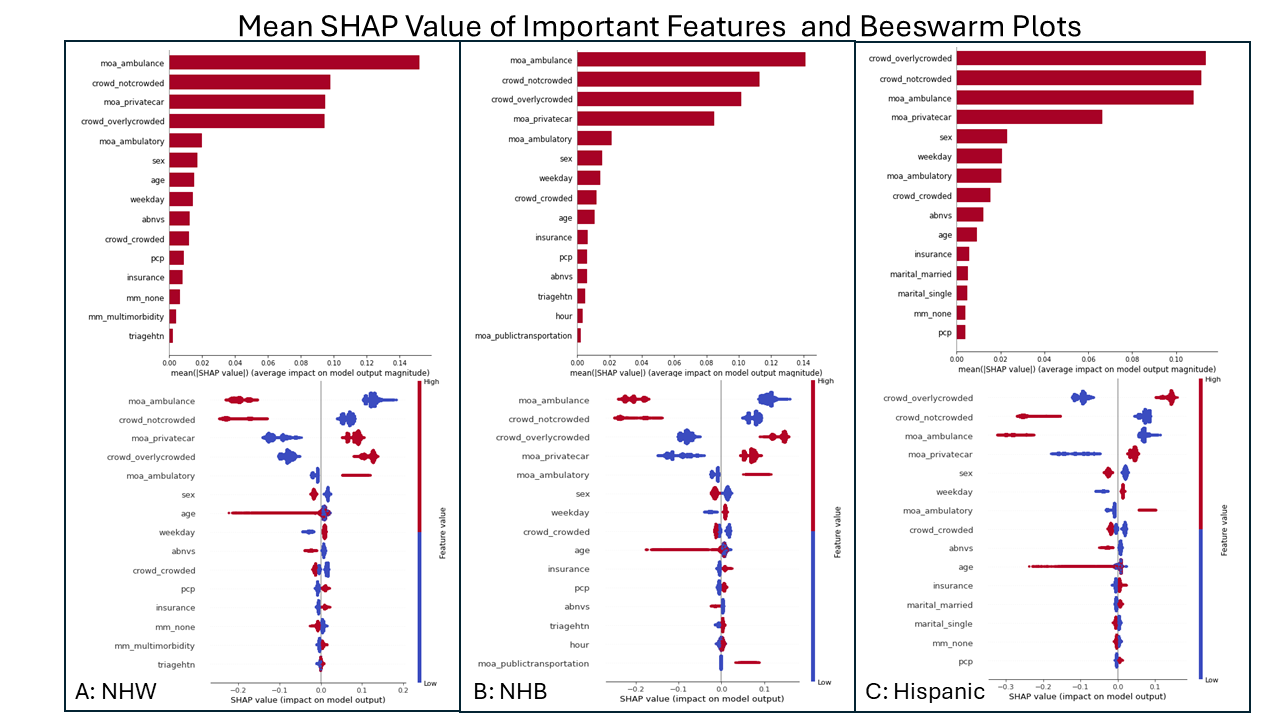

Supplement: S1 Fig — (TIF) [file pdig.0000751.s001.tif]

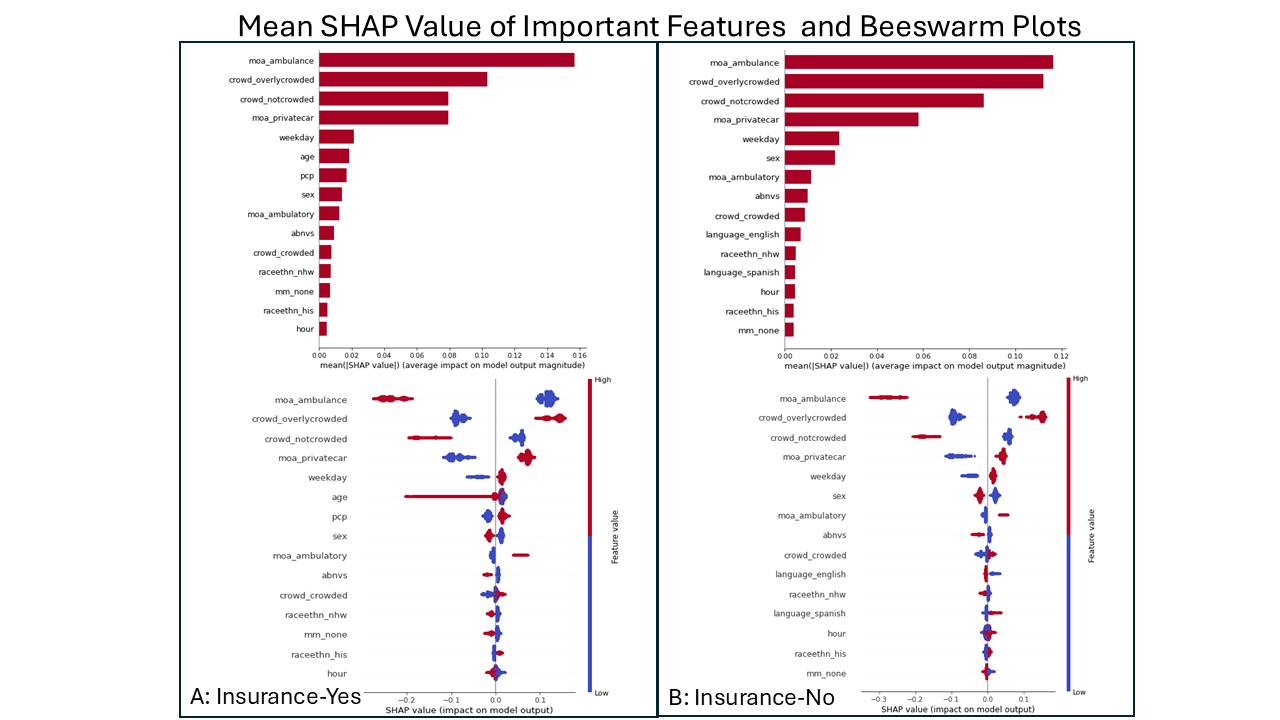

Supplement: S2 Fig — (TIF) [file pdig.0000751.s002.tif]
