# Supplementary material for: Evaluating fairness of machine learning prediction of prolonged wait times in Emergency Department with Interpretable eXtreme gradient boosting
Source: PLOS Digit Health. 2025 Mar 20;4(3):e0000751. doi: 10.1371/journal.pdig.0000751 (PMC11925291; doi:10.1371/journal.pdig.0000751)
Supplement: Attachment — Submitted filename: response (R1).docx [file pdig.0000751.s004.docx]

PDIG-D-24-00431

Evaluating Fairness of Machine Learning Prediction of Prolonged Wait Times in Emergency department with Interpretable eXtreme Gradient Boosting

PLOS Digital Health

Dear Dr. Wang,

Thank you for submitting your manuscript to PLOS Digital Health. After careful consideration, we feel that it has merit but does not fully meet PLOS Digital Health's publication criteria as it currently stands. Therefore, we invite you to submit a revised version of the manuscript that addresses the points raised during the review process.

Please submit your revised manuscript within 60 days Feb 09 2025 11:59PM. If you will need more time than this to complete your revisions, please reply to this message or contact the journal office at digitalhealth@plos.org. When you're ready to submit your revision, log on to https://www.editorialmanager.com/pdig/ and select the 'Submissions Needing Revision' folder to locate your manuscript file.

Please include the following items when submitting your revised manuscript:

* A rebuttal letter that responds to each point raised by the editor and reviewer(s). You should upload this letter as a separate file labeled 'Response to Reviewers'. This file does not need to include responses to any formatting updates and technical items listed in the 'Journal Requirements' section below.

* A marked-up copy of your manuscript that highlights changes made to the original version. You should upload this as a separate file labeled 'Revised Manuscript with Track Changes'.

* An unmarked version of your revised paper without tracked changes. You should upload this as a separate file labeled 'Manuscript'.

If you would like to make changes to your financial disclosure, competing interests statement, or data availability statement, please make these updates within the submission form at the time of resubmission. Guidelines for resubmitting your figure files are available below the reviewer comments at the end of this letter.

We look forward to receiving your revised manuscript.

Kind regards,

Dhiya Al-Jumeily OBE, PhD

Section Editor

PLOS Digital Health

Leo Anthony Celi

Editor-in-Chief

PLOS Digital Health

orcid.org/0000-0001-6712-6626

**Additional Editor Comments (if provided):**

[Note: HTML markup is below. Please do not edit.]

**Reviewers' Comments:**

**Comments to the Author**

Reviewer #1: This work describes the use of ML in predicting wait times for ED patients. This retrospective observational study identified unfairness across protected attributes. I have some comments/suggestions.

Is the dataset that you collected made available to other researchers?
Reply:

The dataset contains restricted data and cannot be made publicly available. If resources permit, we will share the results in open access allowing free distribution under most licenses. (Lines 449-450)

Please try to avoid combining the AI and ML acronyms (line 30), please define individually.
Line 55 reports the number of visits, is it possible to state how many are unique patients?
There are some acronyms such as NHW and NHB which are not defined before they are used (not defined until line 308 but used much earlier).

Reply:

- We have defined AI and ML acronyms individually.
- We have revised the manuscript to include the number of unique patients (99,178 unique patients, Lines 55-56).
- We have provided explanations for the abbreviations "NHW" and "NHB" at their first mention in the manuscript (see Line 103).

Could you describe the distributions of characteristics such as age, gender, and race within the original dataset for example the balance of male vs female in the overall dataset. This is not clear in Table 1. E.g., perhaps add % to the middle row of Figure 1.
Reply:

We revised Figure 1 to describe the distributions of age, gender, race and ethnicity.

Table 2 provides model performance metrics. Are there any alternative methods which could be used to gain comparative results?
Reply:

- We implemented three machine learning algorithms—cross-validated logistic regression (CVLR), random forest (RF), and eXtreme Gradient Boosting (XGBoost). We now include a supplemental table to include all three models.
- However, we retained XGBoost to evaluate fairness because the performance metrics were the same in both training and testing datasets.( Supplemental Table 1).

Line 320 provides a very brief description of the ML algorithm used (XGBoost). Can you justify the choise of XGBoost (did you test other? or does the literature support it?). Can you provide any parameter details or similar to allow reproduction. Are there any abalation studies to support this section?
Reply:

- We revised the manuscript to include hyper-parameters and cross validation used (Lines 365-379).
- The choice of features (covariates) was guided by both practice considerations and scientific literature. We have added references to justify selected features in our study. (Lines 348-350).

References:

1. Guo LL, Guo LY, Li J, et al. Characteristics and admission preferences of pediatric emergency patients and their waiting time prediction using electronic medical record data: retrospective comparative analysis. J Med Internet Res, 2023; 25: e49605. PMID: 37910168.
2. Ataman MG and Sariyer G. Predicting waiting and treatment times in emergency departments using ordinal logistic regression models. Am J Emerge Med, 2021; 46: 45-50. PMID: 33721589.

The conclusion could be improved to highlight the findings of your research and its importance.
Reply:

We now highlight our findings and their importance. (Lines 431-438).

Please edit Figure 2 so that the labels are readable (e.g., increase font size). Similarly, if possible, try to increase the resolution of the image.

Reply: We have revised Figure 2 to increase the resolution and made the labels more readable.

Reviewer #2: 1. Selection bias and sample representativeness emerge from study's focus on ESI-3 patients, excluding ESI-1, 2, 4, and 5 patients. Authors provide rationale for this choice, it limits generalizability of findings and introduces selection bias. This inclusion misses patterns in wait time disparities across acuity levels, providing picture of emergency department dynamics.

Reply:

The rationale for this inclusion is rooted in clinical significance and practice considerations (example: workflow management).

- Generally, patients assigned Emergency Severity Index (ESI) levels 1 and 2 are categorized as having life threatening or emergent conditions requiring immediate attention by healthcare providers. These patients typically experience minimal wait times and rarely leave ED before being seen. As demonstrated in the table below, their average wait time is 1.6 minutes.
- In contrast, patients assigned ESI levels 4 and 5 are considered to have non-emergent or less critical conditions. These patients rely on ED as their primary source of care and comprise less than 10% of total ED visits. Our institution, a publicly funded hospital, has a dedicated fast-track area staffed by Advanced Practice Providers (APPs), who primarily manage patients with ESI levels 4 and 5 and these patients can bypass the main ED. Given this unique ED workflow, we did not include ESI levels 4 & 5 in our analysis.
- The table below summarizes the percentage with prolonged ED wait times and average wait time by ESI-level categories.

| Table: Percent with Prolonged Wait time and Average Wait Time in Minutes by ESI-Level | | | |
| --- | --- | --- | --- |
|  | Percentage | Wait time≥30min | Average wait time (min)  Median [IQR] |
| ESI-1 | 4.00% | 0.07% | 1.6 [0.4-4.2] |
| ESI-2 | 24.01% | 8.10% | 5.3 [3.0-11.3] |
| ESI-3 | 62.57% | 80.14% | 27.5 [5.8-92.4] |
| ESI-4 | 8.46% | 10.85% | 34.3 [13.3-80.3] |
| ESI-5 | 0.96% | 0.85% | 26.0 [9.8-64.0] |
| Total | 100% | 100% | 13.5 [4.1-62.8] |

2. Study's approach to defining wait times presents limitation. Binary categorization of wait times (<30 minutes vs 30 minutes or more) is simplistic. While 30-minute cutoff stems from recommendations, threshold masks variations in wait times. Approach to analyzing wait times as continuous variable or using categories might provide insights into wait time patterns and disparities.

Reply: We revised our manuscript with the followings (Lines 325-343).

**Use of a threshold approach:** We acknowledge that collapsing wait times into two categories (< 30 minutes vs ≥ 30 minutes) can mask important variations.  However, we selected a threshold-based approach to 1) guide decision makers, 2) enable consistent comparison across studies, and 3) assess policy goals towards reducing health disparities. With respect to decision making, a single, clear threshold provides an immediate and straightforward benchmark for hospital administrators, clinicians, and policymakers to guide decision-making.  It allows for rapid identification of patients at risk of prolonged wait times and simplifies performance tracking, quality improvement initiatives, and resource allocation.  In terms of comparisons, using a categorical cutoff makes it easier to compare ED performance across institutions and over time. It facilitates external benchmarking and helps communicate results to stakeholders who may not be familiar with more complex statistical measures.  In terms of policy metrics, the U.S. Department of Health and Human Services, through its *Healthy People 2030* initiative, advocates for reducing the proportion of ED patients experiencing prolonged wait times, aiming for a 12% reduction in such cases. This further underscores the necessity of establishing a categorical ED wait time.

**Selection of 30-minute cut off point:**

Our decision to adopt a 30-minute cut off as prolonged wait time was informed by three key considerations: 1) professional consensus, 2) empirical evidence, and 3) clinical operations management.

The professional consensus emerges from national surveys and recognized wait time standards.  For example, A national survey of academic emergency physicians recommended that the average ED wait time should ideally be less than 30 minutes. Additionally, *Emergency Physician Monthly*, a journal dedicated to emergency medicine professionals, characterized a wait time of under 30 minutes as an “excellent” standard.

From an empirical standpoint, the U.S. Government Accountability Office, reported that the median wait time for ESI-3 patients ranged between 15 and 60 minutes.  The National Center for Health Statistics data reported a median of 30 minutes (2006 National Center for Health Statistics data) in the US. These data support the 30‑minute mark as both clinically relevant and practically achievable in many ED settings.

Furthermore, clinical operations management can be supported by adopting a 30‑minute cutoff because it has the potential to align with operational targets set by emergency medicine professionals and enables EDs to focus on interventions such as triage optimization and patient flow improvements - that help minimize wait times.  This can result in efficient resource utilization, better patient outcomes, and satisfied patients.

References:

1.        Survey by AAAEM (Academy of Administrators in Academic Emergency Medicine) [<https://www.saem.org/about-saem/academies-interest-group-affiliate/aaaem/benchmark-survey2>]

2.        Reiter M. 11 Benchmarks that should matter to EPs. Emergency Physicians Monthly.  [https://epmonthly.com/article/11-benchmarks-that-should-matters-to-eps.]

3.        Hospital Emergency Department: Crowding Continues to Occur, and Some Patients Wait Longer than Recommended Time Frames. [https://www.gao.gov/assets/gao-09-347.pdf]

4.        US HHS Healthy People 2030: Reduce the proportion of emergency department visits with a longer wait time than recommended — AHS‑09. [https://odphp.health.gov/healthypeople/objectives-and-data/browse-objectives/health-care-access-and-quality/reduce-proportion-emergency-department-visits-longer-wait-time-recommended-ahs-09/data#data-chart]

3. Feature engineering in study shows limitations in handling of comorbidities. Categorization into "no chronic condition," "one chronic condition," or "multiple conditions" fails to account for severity or types of conditions, which impact wait times. Categorization misses interaction effects between comorbidities and variables, overlooking relationships that influence wait times.

Reply: We greatly appreciate the reviewer’s insightful comments. The decision to classify patients’ chronic conditions into three categories was based on the following considerations:

1. The current policy task force on multiple chronic conditions strategic framework endorsed by HHS. It is recommended that research be focused on individuals with multiple chronic conditions. Therefore, our study classified patients with “no chronic condition”, “one chronic condition” and “multiple chronic conditions”. [references 1-3]
2. Numerous studies have employed similar classifications for chronic conditions, providing a precedent for this approach (references 4-5).
3. In preliminary analysis we included 16 distinct chronic conditions. These analyses revealed that none of these chronic conditions ranked among the top five features contributing to prolonged wait time prediction.

Given these factors, we opted to categorize chronic conditions into three overarching groups.

References:

1. Goodman RA, Posner SF, Huang ES, et al. Defining and measuring chronic conditions: imperatives for research, policy, program, and practice. Prev Chronic Dis, 2013; 10:E66. PMID: 23618546.
2. Satariano WA and Boyd CM. Improving the evidence base on multimorbidities through better research: a commentary on the U.S. HHS initiative, Multiple Chronic Conditions: A Strategic Framework. J Comorb, 2013; 3(Spec Issue): 18-21. PMID: 29090142.
3. U.S. Department of Health & Human Services. Multiple chronic conditions: A strategic framework. Optimum health and quality of life for individuals with multiple chronic conditions. Washington, DC: U.S. Department of Health & Human Services; 2010. Available from: <http://www.hhs.gov/ash/initiatives/mcc/mcc_framework.pdf> [Last accessed Dec 23, 2024]
4. Yang S, Gao T, Li J, et al. Chronic condition change and its longitudinal association with healthcare utilization among rural older adults: intergenerational financial support as a possible moderator? BMC Geriatr, 2024; 24(1): 837. PMID: 39407124
5. Azubuike CD and Alawode OA. Delayed healthcare due to cost among adults with multimorbidity in the United States. Healthcare (Basel), 2024; 12(22): 2271. PMID: 39595468.

4. Study's model validation raises concerns. Authors mention cross-validation, they don't specify validation strategy or number of folds used, making it hard to assess robustness of methodology. There's no mention of testing for multicollinearity among predictors, which affects model's stability. Study doesn't address temporal autocorrelation, despite using time-series data from 2019 to 2021, which influences reliability of results.

Reply: Thank you for highlighting these important considerations.

- We have revised our manuscript to include detailed descriptions of hyperparameter tuning and cross-validation procedures (Lines 365-379).
- We implemented measures to mitigate potential multicollinearity by examining a heat-map (correlation matrix of all features), carefully selecting features based on clinical significance, practice relevance, and prior research (see references). Additionally, we have updated the Method section with relevant references to strengthen the contextual grounding of our approach (Lines 348-350). Furthermore, we employed regularization techniques, such as Lasso regression, to enhance model stability, which indirectly addresses issues related to multicollinearity. Supporting results for this approach, which were not included in the main manuscript, are listed below.

Regarding temporal autocorrelation, our primary focus was on evaluating the contribution of predictors to the model rather than on forecasting. Nonetheless, we acknowledge that temporal autocorrelation could influence the reliability of results in time-series data. Future studies could address this by incorporating explicit time-series modeling techniques (e.g., ARIMA). We greatly appreciate the insightful feedback and have revised the manuscript to further address this limitation in the Discussion section (Lines 283-286).

References:

1. Guo LL, Guo LY, Li J, et al. Characteristics and admission preferences of pediatric emergency patients and their waiting time prediction using electronic medical record data: retrospective comparative analysis. J Med Internet Res, 2023; 25: e49605. PMID: 37910168.
2. Ataman MG and Sariyer G. Predicting waiting and treatment times in emergency departments using ordinal logistic regression models. Am J Emerge Med, 2021; 46: 45-50. PMID: 33721589.

Preliminary results:

Selected features as the followings: (using R scripts)

X <- model.matrix(wait30 ~ age+raceethn2+raceethn1+raceethn3+raceethn4+raceethn5+marital1+marital2+marital3+moa1+moa2+moa3+moa4+language2+language1+language3+crowd1+crowd2+crowd3+mm0+mm1+mm2+ insurance1+ abnvs1+ weekday1 + sex1+hour1+pcp1+triagehtn1,

+ data = data)	# Predictor matrix (removes intercept)

y <- data$wait30	# Outcome variable

\# Fit Lasso regression

lasso_model <- glmnet(X, y, family = "binomial", alpha = 1)

\# Plot coefficients against log(lambda)

plot(lasso_model, xvar = "lambda", label = TRUE)

title("Lasso Coefficients")

\# Cross-validation to find optimal lambda

cv_model <- cv.glmnet(X, y, family = "binomial", alpha = 1)

optimal_lambda <- cv_model$lambda.min

\# Refit model at optimal lambda

final_model <- glmnet(X, y, family = "binomial", alpha = 1, lambda = optimal_lambda)

\# Extract the coefficients from the model

coefficients <- coef(final_model)

non_zero_features <- rownames(coefficients)[which(coefficients != 0)]

non_zero_features <- non_zero_features[non_zero_features != "(Intercept)"] # Exclude intercept

\# Print selected features

> cat("Selected features at optimal lambda:", paste(non_zero_features, collapse = ", "), "\n")

Selected features at optimal lambda:

age, raceethn2, raceethn1, marital1, moa1, moa2, moa3, language1, language3, crowd1, crowd3, mm0, mm2, insurance1, abnvs1, weekday1, sex1, pcp1, triagehtn1

We performed the XGBoost with the use of these selected features including: age, NHW, NHB, single, moa_ambulance, moa_ambulatory, moa_private_car, language_English, Language_others, not_crowded, overly_crowded, no_chronic_condition, multimorbidity, insurance, abnvs, weekday, sex, pcp, and triagehtn.

5.Study's approach to fairness metrics reveals limitations. It focuses on False Negative Rate (FNR) for clinical significance, it doesn't justify why this metric matters more than others in context of emergency department wait times. Fairness thresholds (0.1 for differences and 0.8-1.2 for ratios) appear arbitrary without justification. Counterfactual analysis doesn't account for interactions between protected attributes, missing intersectionality effects that matter for understanding disparities in wait times.

Reply: The thresholds of 0.1 for differences and 0.8-1.2 for ratios are rules-of-thumb based on historical and practical perspectives.  Historically, the uniform guidelines prohibit employment practices which discriminate on the grounds of race, color, religion, sex, and national origin (Reference 1). The guidelines introduced the “80% rule” or “four-fifths rule” for detecting potential disparate impact in selection processes.  Under this rule, “a selection rate for any race, sex, or ethnic group which is less than four-fifths (4⁄5) or (80%) of the rate for the group with the highest rate” is evidence of adverse impact.

 While 0.80 or (80%) rule identifies when a group is disadvantaged, 1.2 (or 120%) is often used as the upper bound to flag when a group may be receiving disproportionately favorable outcomes. This symmetric range (0.80–1.2) helps ensure fairness is evaluated symmetrically in both directions. Although the 1.2 threshold is not grounded in legal standards, having an upper bound balances health equity consideration.

Although originally designed for employment contexts, this threshold has been widely used in ML fairness research (references 2-5) and incorporated in IBM’s AI Fairness 360 tool (reference 6). While these numbers may be considered arbitrary (reference 7), they provide well-established reference points for consistent measurement and comparison of fairness across diverse contexts.

In this study, we emphasized minimizing FNR due to clinical significance. We explained the rationale of focusing on FNR both in the Discussion and Results sections.

In the Discussion section: (Lines 238-247) “When further interpreting performance accuracy, we prioritized minimizing the FNR due to its critical implications for patient care, where higher FNR could result in fewer predictions of patients experiencing prolonged wait times, potentially delaying their access to healthcare providers and increasing the risk of suboptimal clinical outcomes. For example, in the case of a patient presenting to the ED with a chief complaint of acute abdominal pain, if the patient were experiencing acute appendicitis, and the model predicted a wait time of less than 30 minutes when the actual wait time exceeded 30 minutes (a false negative). Thus, a false negative can delay surgical consultation, which could lead to appendiceal perforation and sepsis, ultimately leading to a prolonged hospitalization and worse clinical outcomes.”

In the Results section: (Lines 144-148) “The model predicted higher FPR (33% vs. 26%) and lower FNR (18% vs. 24%) in females when compared to male individuals. However, in clinical practice, greater emphasis is placed on minimizing the FNR, indicating cases where patients experienced prolonged wait times despite the model predicting otherwise.”

In terms of the counterfactual analysis not including the interactions, we appreciate the reviewer’s raising this important point. We acknowledge that our counterfactual analysis did not explicitly account for interactions between protected attributes, which could reveal intersectional effects critical for understanding disparities in wait times. In this study, our primary focus was on evaluating the main effects of individual attributes to provide an overall assessment of disparities. While this approach simplifies the analysis, we recognize that intersectionality can play a significant role in shaping outcomes. We revised our manuscript with the inclusion of such limitation in the Discussion section (Lines 283-286).

References:

1. Code of Federal Regulations: PART 60-3 Uniform guidelines on employee selection procedures (1978). [<https://www.ecfr.gov/current/title-41/subtitle-B/chapter-60/part-60-3>]
2. Feldman M, Friedler SA, Moeller J, et al. Certifying and removing disparate impact. [KDD '15: Proceedings of the 21th ACM SIGKDD International Conference on Knowledge Discovery and Data Mining](https://dl.acm.org/doi/proceedings/10.1145/2783258). Pages 259 – 268. <https://doi.org/10.1145/2783258.2783311>
3. Kehrenberg T, Chen Z, and Quadrianto N. Tuning fairness by balancing target labels. Front Artif Intell, 2020; 3: 33. PMID: 33733151.
4. Verma S, and Rubin J. Fairness definitions explained. 2018 ACM/IEEE International Workshop on Software Fairness. <https://fairware.cs.umass.edu/papers/Verma.pdf>
5. Barocas S, and Selbst AD. Big Data’s Disparate Impact. California Law Review 671, 2016. Available at: <https://papers.ssrn.com/sol3/papers.cfm?abstract_id=2477899>
6. Trusted AI/AIF 360. Available at: <https://github.com/Trusted-AI/AIF360>
7. Caton S, and Haas C. Fairness in machine learning: A survey. ACM Comput Surv, 2024; 56: 7. Article 166. Available at: <https://dl.acm.org/doi/pdf/10.1145/3616865>

Reviewer #3: The introduction section is too narrow and needs addressing broader aspects of AL/ML use in the healthcare. I would recommend to refer the following studies in healthcare recently (Line 72-73).
<https://royalsocietypublishing.org/doi/full/10.1098/rsos.201823>
<https://www.nature.com/articles/s41598-024-51985-w>
Further highlight the contributions in the Introduction section

Reply: Yes, we revised our introduction with the inclusion of recommended studies in healthcare and highlighted their contributions. (Lines 73-79)

Line 98, ‘we observed more…’. Add specific statistical outcomes/evidence rather than descriptive only.

Reply: Yes, we revised the results with the inclusion of statistical results in these sentences. (Lines 105-107)

Table 1 outcomes are important however showing no variations in the p-value. Thus all of the factors seems associated strongly.

Reply: Yes, it is very common to have statistically significant differences when comparing two groups with large sample size.

Method: Firstly, I would like to see the detailed explanation of the dataset. Ethical considerations even if it is secondary data.
How the dataset is partitioned? Are there repetitive instances? E.g., multiple records per person?

Reply:

- The unit of analysis for our prediction models was an ED visit. There were 173,856 patient ED visits during the study period, made by 99,178 unique patients. Our revised text adds clarity (Line 55).
- We also included percentage of male/female, race/ethnicity, and insurance status in Figure 1.
- As stated in the methods section (Lines 367-368), the data were partitioned into training (70%) and testing (30%) datasets.

How did you chose 30 minute to categorize the data? I would like to see support for this e.g., literature

Reply: We revised our manuscript with the following explanations and added more references (Lines 325-343).

**Use of a threshold approach:** We acknowledge that collapsing wait times into two categories (< 30 minutes vs ≥ 30 minutes) can mask important variations.  However, we selected a threshold-based approach to 1) guide decision makers, 2) enable consistent comparison across studies, and 3) assess policy goals towards reducing health disparities. With respect to decision making, a single, clear threshold provides an immediate and straightforward benchmark for hospital administrators, clinicians, and policymakers to guide decision-making.  It allows for rapid identification of patients at risk of prolonged wait times and simplifies performance tracking, quality improvement initiatives, and resource allocation.  In terms of comparisons, using a categorical cutoff makes it easier to compare ED performance across institutions and over time. It facilitates external benchmarking and helps communicate results to stakeholders who may not be familiar with more complex statistical measures.  In terms of policy metrics, the U.S. Department of Health and Human Services, through its *Healthy People 2030* initiative, advocates for reducing the proportion of ED patients experiencing prolonged wait times, aiming for a 12% reduction in such cases. This further underscores the necessity of establishing a categorical ED wait time.

**Selection of 30-minute cut off point:**

Our decision to adopt a 30-minute cut off as prolonged wait time was informed by three key considerations: 1) professional consensus, 2) empirical evidence, and 3) clinical operations management.

The professional consensus emerges from national surveys and recognized wait time standards.  For example, A national survey of academic emergency physicians recommended that the average ED wait time should ideally be less than 30 minutes. Additionally, *Emergency Physician Monthly*, a journal dedicated to emergency medicine professionals, characterized a wait time of under 30 minutes as an “excellent” standard.

From an empirical standpoint, the U.S. Government Accountability Office, reported that the median wait time for ESI-3 patients ranged between 15 and 60 minutes.  The National Center for Health Statistics data reported a median of 30 minutes (2006 National Center for Health Statistics data) in the US. These data support the 30‑minute mark as both clinically relevant and practically achievable in many ED settings.

Furthermore, clinical operations management can be supported by adopting a 30‑minute cutoff because it has the potential to align with operational targets set by emergency medicine professionals and enables EDs to focus on interventions such as triage optimization and patient flow improvements - that help minimize wait times.  This can result in efficient resource utilization, better patient outcomes, and satisfied patients.

References:

1.        Survey by AAAEM (Academy of Administrators in Academic Emergency Medicine) [<https://www.saem.org/about-saem/academies-interest-group-affiliate/aaaem/benchmark-survey2>]

2.        Reiter M. 11 Benchmarks that should matter to EPs. Emergency Physicians Monthly.  [https://epmonthly.com/article/11-benchmarks-that-should-matters-to-eps.]

3.        Hospital Emergency Department: Crowding Continues to Occur, and Some Patients Wait Longer than Recommended Time Frames. [https://www.gao.gov/assets/gao-09-347.pdf]

4.        US HHS Healthy People 2030: Reduce the proportion of emergency department visits with a longer wait time than recommended — AHS‑09. [https://odphp.health.gov/healthypeople/objectives-and-data/browse-objectives/health-care-access-and-quality/reduce-proportion-emergency-department-visits-longer-wait-time-recommended-ahs-09/data#data-chart]

How the categorical data and numerical data are transformed for the ML? e.g., nominal or ordinal data

Reply: In this study, categorical data are transformed as nominal data.

Limitations of the study should be addressed
Better to explain ML algorithm in the context of this study

Reply: Yes, we revised our limitation in our Discussion section. Meanwhile, we expanded our ML algorithm to address more detail on how to conduct XGBoost in this study (Lines 365-379).

Reviewer #4: This is a nice example of examining a ML model for fairness across protected attribute groups. Below I include minor edits and suggestions, but include more substantial questions/concerns as well.

Major questions/concerns:

Results section, first paragraph: a lot of the text here is redundant and should be cut. The text after the mention of Table 2 (which should be Table 1) reiterates what was just said previously.
Reply: Yes, we revised the result section with the exclusion of redundant sentences. However, Figure 1 mainly illustrates the wait time difference by sex, race/ethnicity, and insurance status, which are the three main domains for fairness evaluation. Therefore, we would like to keep Figure 1.

Lines 143-145: “Overall, in terms of the clinical application focusing on FNR, the XGBoost prediction model exhibited better performance among female patients, Hispanic patients, and patients without insurance coverage, which matched our findings of prolonged wait times occurring more often among patients within these subgroups (Figure 1).”
This doesn’t make sense the way you have written it. FNR is describing a type of error the model is making whereas the higher prevalence of prolonged wait times is referencing the actual data. These are not equivalent or comparable in this context. I *think* you may be trying to describe the calibration of the model but I’m not sure.

Reply: Sorry for the confusion, we deleted the second half of this sentence to make it less confused. (Lines 154-155)

I’m wondering why only XGBoost was used and not multiple models. Training 3-6 different ML models and comparing their performance is pretty standard.

Reply:

- We implemented three machine learning algorithms—cross-validated logistic regression (CVLR), random forest (RF), and eXtreme Gradient Boosting (XGBoost). We now include a supplemental table to include all three models.
- However, we retained XGBoost to evaluate fairness because the performance metrics were the same in both training and testing datasets.( Supplemental Table 1).

It would be more compelling to also investigate if any tweaks to the ML model(s) that might reduce or mitigate the bias found.

Reply:

- We revised the manuscript to include hyper-parameters and cross validation used (Lines 365-379).
- The choice of features (covariates) was guided by both practice considerations and scientific literature. We have added references to justify selected features in our study. (Lines 348-350).

References:

1. Guo LL, Guo LY, Li J, et al. Characteristics and admission preferences of pediatric emergency patients and their waiting time prediction using electronic medical record data: retrospective comparative analysis. J Med Internet Res, 2023; 25: e49605. PMID: 37910168.
2. Ataman MG and Sariyer G. Predicting waiting and treatment times in emergency departments using ordinal logistic regression models. Am J Emerge Med, 2021; 46: 45-50. PMID: 33721589.

Lines 261 and 262: “Additionally, not all potential confounders were
considered in the training of the prediction model.” - These are not considered confounders since this is a prediction model and not a causal model. This term should be replaced with “predictors”.
Reply: Yes, we revised it. (Line 277)

Minor edits:

Throughout: gender should be called sex.
Reply: Yes, we revised it. (see track changes)

Introduction section, second paragraph: What does “overall predictions” mean here? It would be clearer to be more specific.
Reply: Yes, we revised this sentence to be more specific to “overall performance accuracy” (Lines 84-85).

Introduction section, third paragraph: I would edit this sentence: “Upon identifying unfairness, appropriate adjustments can be made to the ML model predictions to enhance their accuracy and clinical applications.” to something like: “Upon identifying unfairness, appropriate adjustments made to the ML model may enhance the accuracy of the predictions and fairness in clinical applications.” I suggest this because it isn’t always possible to correct for bias (unfairness) by altering the model in some way; sometimes having more data or better quality data is the only way to improve model performance/fairness.
Reply: Yes, we revised it accordingly. (Lines 90-92)

Results section, first sentence (line 97)- need to define NHW and NHB first time you use them in the text, e.g., Non-Hispanic White (NHW).
Reply: Yes, we revised it accordingly. (Lines 104-105)

Results section, first paragraph minor typo (line 99): “We observed that more ED visits from females, Hispanics, and patients without insurance tended to have a prolonged wait times that those from males” - “that” should be “than”.
Reply: Yes, we revised it accordingly. (Line 107)

Results section, first paragraph (line 100): ”…and patients with
insurance, as illustrated in Figure 1.” - I’m wondering what the utility of Figure 1 is since that information is presented in Table 1. I would remove Figure 1 from the manuscript and point the reader to Table 1.
Reply: Yes, we revised the result section with the exclusion of redundant sentences. However, Figure 1 mainly illustrates the wait time difference by sex, race/ethnicity, and insurance status, which are the three main domains for fairness evaluation. Therefore, we would like to keep Figure 1.

Results section, first paragraph: “General characteristics of the study patients are listed in Table 2.” - it should be Table 1
Reply: Yes, we revised it accordingly. (Line 109)

The quality of Figure 2 needs to be improved.
Reply: Yes, we revised it accordingly.

General comment: all of the text needs to be reviewed for grammatical errors - there are a multitude of them.
Reply: Yes, we have provided grammatical revisions throughout the entire manuscript.

**Figure resubmission:**

While revising your submission, please upload your figure files to the Preflight Analysis and Conversion Engine (PACE) digital diagnostic tool, https://pacev2.apexcovantage.com/. PACE helps ensure that figures meet PLOS requirements. To use PACE, you must first register as a user. Registration is free. Then, login and navigate to the UPLOAD tab, where you will find detailed instructions on how to use the tool. If you encounter any issues or have any questions when using PACE, please email PLOS at figures@plos.org. Please note that Supporting Information files do not need this step. If there are other versions of figure files still present in your submission file inventory at resubmission, please replace them with the PACE-processed versions.

**Reproducibility:**

To enhance the reproducibility of your results, we recommend that authors of applicable studies deposit laboratory protocols in protocols.io, where a protocol can be assigned its own identifier (DOI) such that it can be cited independently in the future. Additionally, PLOS ONE offers an option to publish peer-reviewed clinical study protocols. Read more information on sharing protocols at https://plos.org/protocols?utm_medium=editorial-email&utm_source=authorletters&utm_campaign=protocols
